# Can Patients with Hematologic Disease and Prior Mucormycosis Undergo Stem Cell Transplantation?

**DOI:** 10.3390/jof12060423

**Published:** 2026-06-11

**Authors:** Armando Leon, Rachel S. Hicklen, Ying Jiang, Adam G. Stewart, Sebastian Wurster, Dimitrios P. Kontoyiannis

**Affiliations:** 1 Department of Infectious Diseases, Infection Control and Employee Health, The University of Texas MD Anderson Cancer Center, Houston, TX 77030, USA; arleon1@mdanderson.org (A.L.); yijiang@mdanderson.org (Y.J.); agstewart@mdanderson.org (A.G.S.); stwurster@mdanderson.org (S.W.); 2 Section of Infectious Diseases, Department of Medicine, Baylor College of Medicine, Houston, TX 77030, USA; 3 Research Medical Library, The University of Texas MD Anderson Cancer Center, Houston, TX 77030, USA; rshicklen@mdanderson.org

**Keywords:** mucormycosis, Mucorales, stem cell transplant, hematologic malignancy, immunocompromised host

## Abstract

The prognosis of mucormycosis after hematopoietic stem cell transplantation (HSCT) is generally poor but data on post-HSCT outcomes in patients with pre-HSCT mucormycosis are limited. We reviewed patients with documented mucormycosis at MD Anderson Cancer Center (2008–2024) and identified five patients who subsequently underwent HSCT. A literature review identified 24 additional such cases. Most patients had acute myeloid leukemia (69%). The most common site of mucormycosis was pulmonary (59%), while 31% had disseminated mucormycosis. All patients received antifungals and 76% had surgery prior to HSCT. At the time of HSCT, 67% had mucormycosis responding to treatment. No patient went to transplant with progressing mucormycosis. Eighty percent of patients with ≥12 months of follow-up after HSCT were alive. Five of the twenty-nine patients (17%) had documented or suspected mucormycosis recurrence post-HSCT. Relapsed malignancy pre-HSCT was associated with increased 12-month post-HSCT mortality (*p* = 0.031). Furthermore, post-transplant mortality was higher in cord blood recipients (*p* = 0.019) and tended to be higher in patients not undergoing surgery pre-HSCT (*p* = 0.062). Despite publication bias, our data suggest that HSCT can be conducted safely in selected patients with pre-HSCT mucormycosis, particularly when underlying hematologic malignancy is in remission, mucormycosis is stable, and surgical source control is feasible.

## 1. Introduction

Since the initiation of routine mold-active prophylaxis for patients with hematologic malignancies and hematopoietic stem cell transplantation (HSCT) with neutropenia, non-*Aspergillus* mold infections (NAIMI) have been increasingly encountered in clinical practice. Mucormycosis (MCM) is the most common NAIMI [1], and it has historically been associated with high mortality, especially in patients with disseminated disease and/or persistent immune compromise [2].

Several studies have investigated outcomes of HSCT recipients with post-transplant MCM, showing median survival times of under two months and an overall MCM-related mortality rate of around 75% (range between 36% and 91% in various studies) [3,4,5]. In contrast to MCM occurring post-HSCT, data on post-transplant outcomes and their determinants in patients with pre-transplant MCM are scarce.

Guidelines recently published by the American Society of Transplantation and Cellular Therapy for the management and prevention of NAIMI in hematopoietic cell transplantation provided general recommendations for transplantation of patients with a history of NAIMI [6]. The authors highlighted factors that may lead to better outcomes such as evidence of at least partial response to antifungal therapy, surgical debulking of infectious foci, remission of hematologic malignancy, and preference of peripheral blood stem cell grafts over bone marrow due to more rapid neutrophil engraftment [6]. They also cautioned about higher risk of post-transplant NAIMI recurrence in patients with active hematologic malignancy, prolonged cytopenias, severe or refractory graft-versus-host-disease (GVHD), and disseminated infections.

In light of ongoing advances in antifungal therapy and the improvement in supportive care and monitoring strategies after stem cell transplant, we reviewed both our experience at MD Anderson Cancer Center (MDACC) and published cases of patients undergoing HSCT with a pre-transplant history of MCM. Specifically, we herein present an illustrative case, characterize post-transplant outcomes and risk of MCM recurrence, determine patient characteristics and variables related to MCM management that are associated with post-transplant mortality, and identify commonalities of cases with favorable outcomes.

## 2. Methods

### 2.1. Case Identification

Our analysis included patients with documented MCM (confirmed by histopathology, microbiology, or molecular testing) prior to undergoing HSCT. Cases without microbiological or pathology confirmation of MCM were excluded. No age restrictions were applied.

To identify such cases, we reviewed the medical records of all patients diagnosed with proven MCM between 2008 and 2024 at MDACC and identified 5 patients who underwent subsequent HSCT. We further performed a comprehensive review of the literature published in English by searching Medline (Ovid), Embase (Ovid), Scopus, and Google Scholar with no date restriction until 9 September 2025, using controlled vocabulary and natural language terms for mucormycosis, zygomycosis, mucor, zygomyco*, *Rhizopus*, *Rhizomucor*, hematopoietic stem cell transplantation, stem cell transplantation, bone marrow transplantation, cord blood stem cell transplantation, peripheral blood stem cell transplantation, stem cell, bone marrow, cord blood, allogenic, and allo-genic. A total of 21 relevant articles were obtained and included in this review, with a total of 24 unique cases of pre-HSCT MCM published between 1996 and 2025 [7,8,9,10,11,12,13,14,15,16,17,18,19,20,21,22,23,24,25,26].

### 2.2. Variables and Definitions

We reviewed demographic information (age, sex), underlying hematological conditions, malignancy status, primary and secondary antifungal prophylaxis, MCM infection site, MCM diagnosis, Mucorales genus identified, antifungal therapy, surgical intervention and adjunct therapies, time from MCM to HSCT, conditioning regimen, transplant source and type, comorbidities, post-transplant complications including co-infections and graft-versus-host disease (GVHD), high-dose glucocorticosteroid (GCS) therapy, MCM recurrence post-HSCT, and crude mortality.

Disseminated MCM was defined as an infection involving two noncontiguous sites [5]. Response to MCM therapy was defined using published criteria [27]. GVHD included all grades and organs involved. High-dose GCS exposure was defined as equivalent to prednisone ≥ 20 mg/day for two or more weeks [28]. Myeloablative conditioning regimens were defined as those including standard dosing of busulfan, fludarabine, clofarabine, cyclophosphamide, cladribine, thiotepa, alemtuzumab, treosulfan, etoposide, amsacrine, and/or anti-thymocyte globulin [29].

### 2.3. Statistical Analysis

Kaplan–Meier survival analysis with the Mantel–Cox log-rank test and univariable Cox proportional hazards regression models were used to evaluate the association between clinical variables and 12-month post-transplant mortality. Firth’s penalized partial likelihood was used in the Cox analyses to account for the small number of events, reducing small-sample bias and preventing infinite or unstable estimates. The limited number of 12-month deaths precluded multivariable Cox regression analysis. All statistical tests were two-sided, with a significance level of 0.05. Data analyses were performed using Prism v10.6.1 (GraphPad Software Inc., Boston, MA, USA) and SAS version 9.4 (SAS Institute Inc., Cary, NC, USA).

## 3. Results

### 3.1. Previously Unpublished Cases from MD Anderson Cancer Center

**Figure 1:**
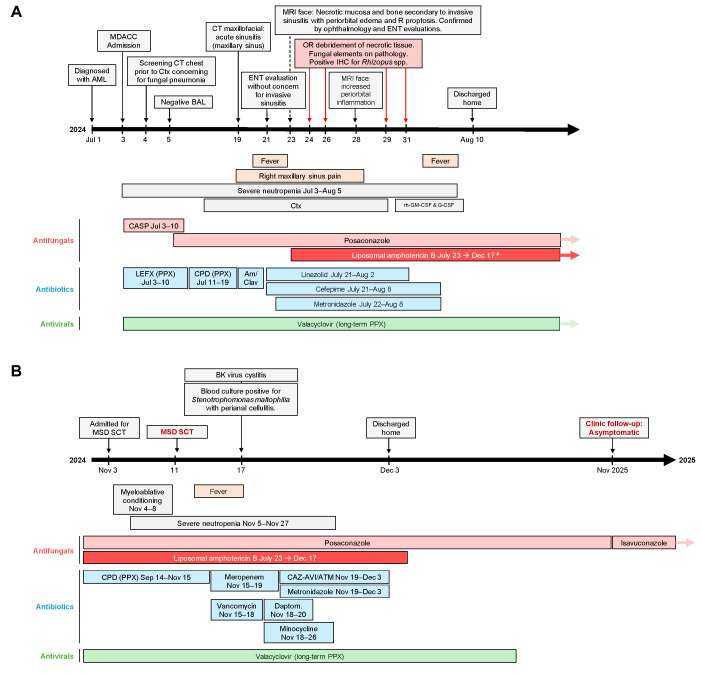
Timeline of clinical events and antimicrobial therapy divided into pre- (**A**) and post-HSCT (**B**) periods. #: Retrobulbar amphotericin B from 07/30 to 08/02. Abbreviations: OR: operating room; MSD (matched sibling donor); CTx: chemotherapy; IHC: immunohistochemistry; ENT: ears, nose, throat; LEFX: levofloxacin; PPX: prophylaxis; Am/Clav: amoxicillin-clavulanate; CPD: cefpodoxime; CAZ-AVI: ceftazidime-avibactam; ATM: aztreonam; Daptom: daptomycin.

**Figure 2:**
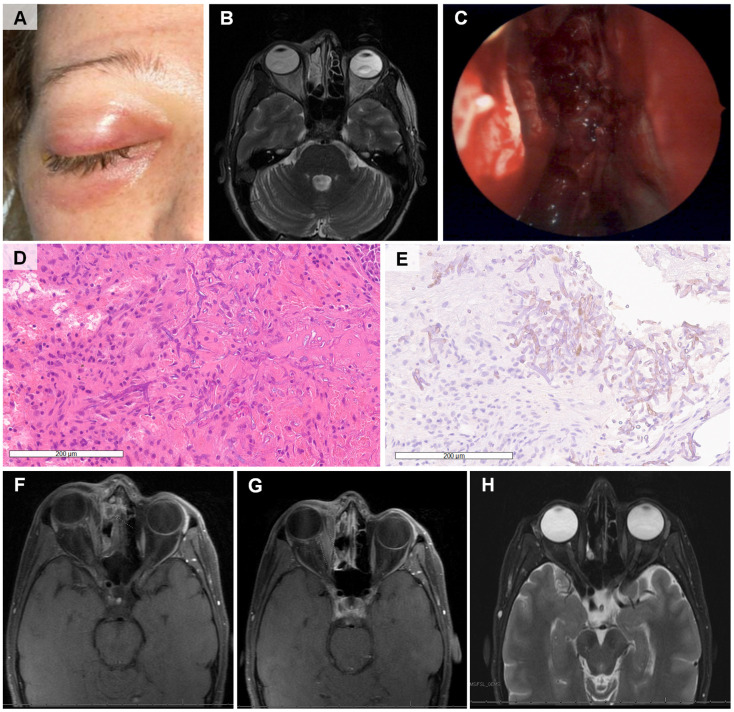
Case presentation. Clinical, radiologic, and histopathology findings. (**A**) Right eye with periorbital edema and swelling. (**B**) MRI face showing right maxillary sinus mucosal thickening and anterior periorbital soft tissue edema with right-sided proptosis. (**C**) Nasal endoscopy showing areas of necrosis over right maxillary sinus. (**D**) Maxillary sinus histopathology (hematoxylin–eosin) with visible non-septate hyphae consistent with *Rhizopus* (20×). (**E**) Maxillary sinus histopathology with immunohistochemistry positive for *Rhizopus* (20×). (**F**) MRI orbits five days after image (**B**) showing non-enhancing T2 hypointense tissue in right anterior ethmoid air cell and frontal and ethmoidal junction concerning for progressing infection. (**G**) Additional image from MRI orbits (**F**) showing increased retrobulbar fat stranding medial and lateral to the medial rectus muscle. (**H**) MRI orbits obtained a year after HSCT showing significant improvement with minimal T2 hyperintense right maxillary sinus mucosa enhancement and post-operative changes without any orbital or periorbital abnormalities.

A representative case report of a 37-year-old female with acute myeloid leukemia (AML) developing sino-orbital mucormycosis and requiring complex management before undergoing HSCT is provided and further illustrated in Figure 1 and Figure 2. Four additional unpublished MD Anderson cases of HSCT being performed after documented MCM are provided in Appendix A.

### 3.2. Case Report

A 37-year-old white female was newly diagnosed with acute myeloid leukemia (AML) started on remission induction chemotherapy with cladribine, cytarabine, and idarubicin (CLIA); quizartinib, and antimicrobial prophylaxis with caspofungin, levofloxacin, and valacyclovir, was initiated (Figure 1).

The patient’s baseline chest CT (computed tomography) was concerning for possible fungal pneumonia with right upper lobe and left lower lobe nodular opacities with ground glass halos; however, bronchoalveolar lavage (BAL) was non-diagnostic. Nonetheless, intravenous (i.v.) caspofungin 50 mg every 24 h was continued and oral posaconazole 300 mg was initiated.

Three weeks into the hospitalization, the patient reported right maxillary pain with CT maxillofacial showing mucosal thickening in the right maxillary sinus with air-fluid level and opacification extending to the right middle meatus consistent with acute sinusitis without osseous erosion. Nasal endoscopy showed boggy middle turbinate with dusky areas in posterior aspect but without signs of invasive fungal sinusitis. However, facial MRI (magnetic resonance imaging) obtained due to persistent symptoms two days after the CT revealed necrotic mucosa and bone erosion secondary to invasive sinusitis as well as edema of the periorbital soft tissue anterior to the maxillary sinus with right-sided proptosis. Repeat nasal endoscopy on the same day confirmed necrotic tissue. On the following day, the patient underwent debridement with biopsy revealing rhino-orbital MCM with immunohistochemistry positive for *Rhizopus* and possibly focal positivity for *Aspergillus* (Figure 2).

The patient was switched to i.v. liposomal amphotericin B (LAmB) 5 mg/kg/day (briefly increased to 7.5 mg/kg/day for 13 days during initial hospitalization), and oral posaconazole was increased to 400 mg/day. In the following week she underwent four sinus debridements due to persistent and slowly progressing rhino-orbital disease, assessed both clinically and by repeat MRIs. Fungal elements were again seen on frozen section. She was also started on daily amphotericin B (AMB) deoxycholate nasal rinses, and ophthalmology performed daily consecutive retrobulbar LAmB injections for three days. In addition, she was administered recombinant human granulocyte-macrophage colony-stimulating factor (rh-GM-CSF) and granulocyte colony-stimulating factor (G-CSF) for seven days with neutrophil recovery after five days of treatment. With eventual recovery of her neutrophil counts and with the aforementioned multiple interventions, she slowly improved and was discharged home. Bone marrow aspiration was consistent with complete remission of AML.

Antifungal therapy was continued for 5 months, with LAmB being switched from daily to three times weekly, then twice weekly, and finally once weekly. She continued on oral posaconazole.

Having been completely asymptomatic for 4 weeks, with significant improvement in imaging and repeat nasal endoscopies, the patient was cleared for HSCT, which was indicated due to her high-risk AML.

Almost 4 months after initial diagnosis of rhino-orbital MCM, and while still on LAmB and posaconazole, she underwent myeloablative conditioning with busulfan, fludarabine, cladribine, and thiotepa and received a matched sibling donor peripheral HSCT with post-HSCT cyclophosphamide.

Her immediate post-HSCT course was complicated by *Stenotrophomonas maltophilia* bacteremia with perianal cellulitis and BK virus cystitis. She engrafted 18 days post-HSCT, and her leukemia remained in remission. Two months after HSCT, she developed stage 2/grade I acute skin GVHD treated successfully with prednisone taper starting at 1 mg/kg/day for one month.

The patient was continued on secondary prophylaxis with oral posaconazole (200 mg every 12 h), whereas LAmB was discontinued 6 weeks after HSCT. Secondary prophylaxis was eventually switched from posaconazole to isavuconazole 12 months after HSCT due to elevated liver enzymes and presumed liver GVHD treated with prednisone. As of today (22 months post-HSCT), there has been no clinical (including repeat nasal endoscopies) or radiological (orbital/sinus MRIs) evidence of MCM recurrence.

### 3.3. Data Review

Combining the data from our five MD Anderson patients and 24 cases from the published literature, we reviewed a total of 29 cases of HSCT being performed after documented MCM. Patient characteristics are summarized in Table 1. The median age of patients was 44 years with nearly even sex distribution. The majority of patients (n = 20; 69%) had AML as their underlying hematologic disease and 26% of patients had relapsed disease at the time of initial diagnosis of MCM.

Only one patient was on Mucorales-active antifungal prophylaxis prior to MCM diagnosis, whereas 16 patients (57%) had received non-Mucorales-active prophylaxis; the remaining 11 patients with information available had not received any antifungal prophylaxis. The most common site of MCM (59%) was pulmonary (including sinopulmonary), while 31% had disseminated disease. Confirmation of MCM diagnosis using overlapping modalities involved histopathology in most patients (72%), followed by culture (31%) and PCR (24%). Mucorales genera identified by PCR and/or culture were predominantly *Rhizopus* and *Lichtheimia* (both 33%), followed by *Rhizomucor* (25%) and *Mucor* (8%).

All patients received AMB-based therapy (including liposomal, lipid complex, and deoxycholate) as part of their MCM therapy and 52% received combination therapy with a Mucorales-active azole. Additionally, 76% of patients underwent surgical interventions for MCM management prior to HSCT. Specifically, twelve (55%) patients underwent pulmonary surgery, five (23%) patients had sinus surgery, and the remaining five (23%) had surgery in other sites (detailed in Table S1, and footnote of Table 1). As part of multimodal management, seven patients (24%) received immunotherapy, including granulocyte colony-stimulating factor (n = 3), granulocyte transfusion (n = 2), or both (n = 2). One patient was treated with hyperbaric oxygen.

All patients had achieved at least stable disease prior to HSCT, while 52% had partial response and 15% had complete response to initial MCM therapy. No patient went to HSCT with progressive MCM.

The median time from initial MCM diagnosis to HSCT was 3 months (range 0–24 months). All patients continued MCM-active secondary prophylaxis at least through the time of HSCT, receiving either only LAmB (n = 14, 48%), posaconazole (n = 5, 17%), isavuconazole (n = 7, 24%), or combined secondary prophylaxis with AMB and MCM-active triazole (n = 3, 10%). The median duration of secondary prophylaxis post-HSCT was 6.5 months, and 25% of patients continued for more than 12 months post-HSCT.

The majority of patients (69%) received myeloablative conditioning, while 31% had reduced-intensity conditioning. While most patients received HSCT for management of their underlying hematologic disease, HSCT was primarily indicated as salvage treatment for MCM in two patients [7,8]. Ninety percent of HSCTs were allogeneic and the majority of those (88%) received peripheral blood transplant. Two patients (8%) received cord blood transplant. Only 17% of HSCTs were haploidentical.

Out of the 26 patients who received allogeneic HSCT, 10 (38%) developed GVHD. Two of them had severe gastrointestinal (GI) involvement, one had severe skin involvement, and one had both. The other six patients had only mild GVHD.

### 3.4. Post-HSCT Outcomes and MCM Recurrence

**Figure 3:**
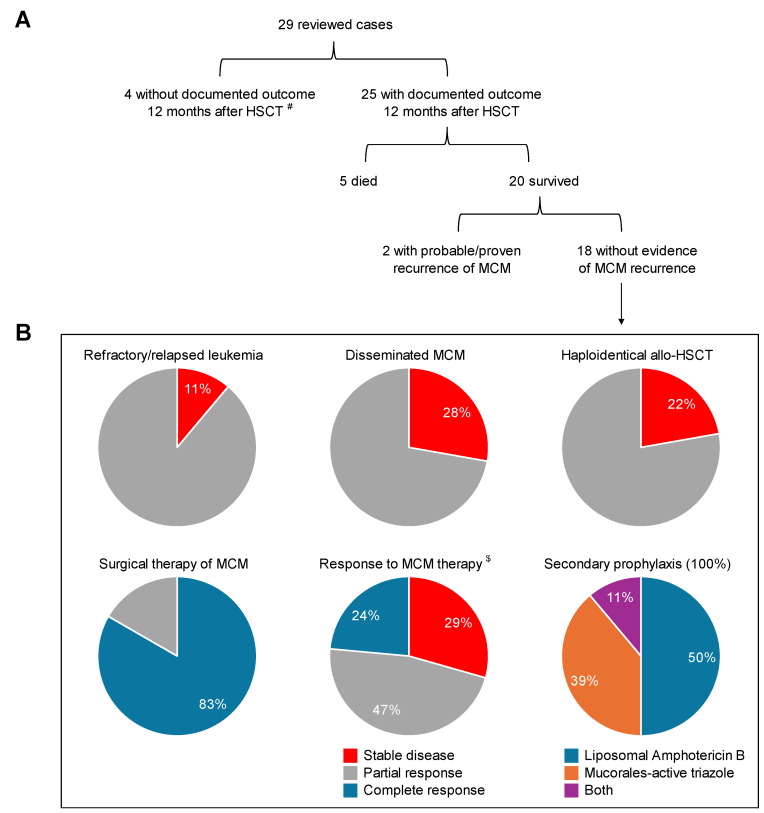
(**A**) Breakdown of outcomes in patients with HSCT post-MCM. (**B**) Key clinical variables among 18 patients who survived 12 months post-HSCT without MCM recurrence. ^#^ A total of 3 patients were censored due to loss to follow-up within 12 months and 1 patient without follow-up duration was also excluded. ^$^ Data not available for one patient.

Twenty-five out of the twenty-nine cases had definitive information on 12-month post-HSCT survival outcomes. Among those, 20 (80%) were alive at 12 months (Figure 3). Notably, only two (10%) of them had probable/proven MCM recurrence, including one who had recurrence of maxillary sinusitis with surgical biopsy confirming MCM and one who had new pneumonia and a lung biopsy consistent with MCM.

Out of the five patients dying prior to 12 months post-HSCT, two were from our institution. Both had at least possible recurrence or progression of fungal pneumonia post-HSCT while still on active triazole secondary prophylaxis. However, both had additional contributing causes of death, including co-infections or progression of their underlying malignancies. One of the other three patients dying within 12 months post-HSCT had possible recurrence of disseminated MCM with new brain lesions but also had severe GVHD and developed interstitial pneumonitis that ultimately led to their demise. The other two patients had no reported signs of MCM recurrence but one presented an idiopathic pulmonary syndrome, and the other one had relapse of their AML, both leading to their respective deaths.

### 3.5. Variables Associated with 12-Month All-Cause Mortality Post-HSCT

**Figure 4:**
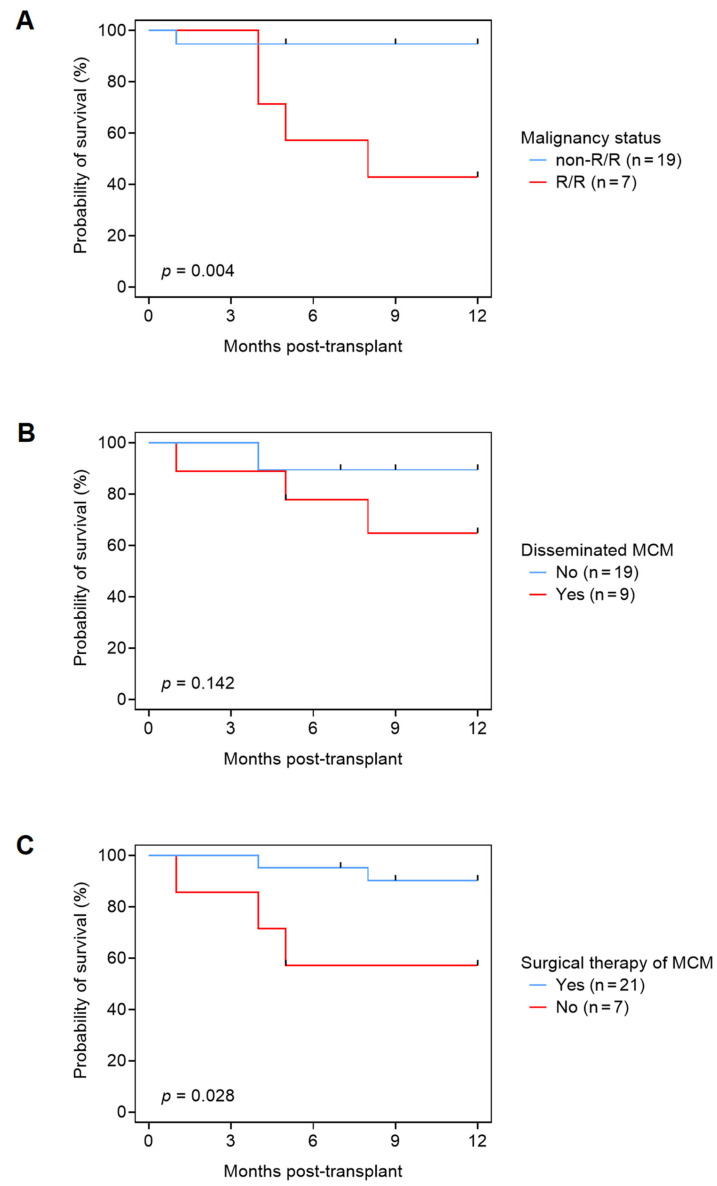
Survival curve analysis evaluating the impact of key oncological and MCM-related pre-transplant variables on 12-month post-transplant mortality. (**A**) Survival curve comparing malignancy status. (**B**) Survival curve comparing disseminated vs localized MCM. (**C**) Survival curve comparing surgical vs nonsurgical therapy of MCM. One patient with unknown follow-up period was excluded. Additionally, two patients with non-malignant diseases were excluded for (**A**). Mantel–Cox log-rank test. Tick marks indicate censored subjects. R/R = relapsed/refractory.

Next, we tested the association of pre-HSCT (including MCM manifestation and management), HSCT-related, and post-HSCT variables with 12-month all-cause mortality post-HSCT (Table 1, Figure 4). Here, refractory or recurrent malignancy at the time of MCM diagnosis in those with underlying leukemia was a strong predictor of poor post-HSCT outcomes (*p* = 0.031). This association was corroborated by survival curve analysis (*p* = 0.004, Figure 4). Furthermore, post-HSCT mortality tended to be higher in patients with disseminated MCM than in those with localized infections, although significance was not reached (*p* = 0.194). Surgical management of MCM was associated with better survival outcomes post-HSCT although it only reached significance on survival curve analysis (*p* = 0.028, Figure 4) but not in Firth’s penalized partial likelihood Cox regression model (*p* = 0.062). Patients who received cord blood transplants had significantly increased mortality when compared to peripheral or bone marrow transplant recipients (*p* = 0.019). Leukemia relapse post-HSCT (*p* = 0.165) showed a trend of being associated with 12-month mortality. Notably, post-HSCT MCM recurrence (possible, probable, or proven) was significantly associated with increased mortality (*p* = 0.016).

### 3.6. Commonalities of Survivors Without Evidence of MCM Recurrence

A total of 18 patients survived for at least 12 months post-HSCT and had no reported evidence of MCM recurrence. As the very small number of survivors with MCM recurrence precluded definitive statistical comparisons by recurrence status, we focused on descriptively identifying pre-HSCT commonalities among the 18 survivors without MCM recurrence (Figure 3). Consistent with the overall cohort, most of them had AML (13/18, 73%), although only two of them (11%) had recurrent or refractory AML. The majority (13/18; 73%) of this group had localized infection (67% pulmonary or sinopulmonary), yet five (28%) had disseminated MCM. Half of these patients received LAmB monotherapy or combination therapy against MCM, respectively. The vast majority (83%) had additional surgical management of MCM. Consistent with the overall cohort, all 17 patients with documented responses had at least stable disease, while 12 (71%) had partial or complete response to MCM therapy. All the patients received secondary prophylaxis.

## 4. Discussion

The decision to proceed with HSCT in patients with a history of MCM has been a controversial subject as the published data are far from robust and essentially anecdotal. This study aimed to address this gap in knowledge by combining a review of our institutional experience and a literature review. Reassuringly, we found positive outcomes for the majority of patients, with 69% surviving through their respective follow-up periods and an only 17% recurrence rate of MCM post-HSCT. However, that relapse rate of mucormycosis post-HSCT in our selected data set appeared higher to the ones reported for aspergillosis in recent prospective studies [30] in patients with acute leukemia (around 5%), while no comparative data between relapse rates of mucormycosis and aspergillosis exist. In addition, clinical studies of relapse of mold infection post-HSCT lack the genetic profiling of culture-documented cases. Therefore, one cannot differentiate reinfection from relapse of infection.

However, several factors related to MCM management and underlying host variables showed associations with post-HSCT outcomes and might inform patient selection for HSCT. For instance, recently published guidelines for the management and prevention of NAIMI in HSCT recipients suggested worse outcomes in patients with relapsed hematologic malignancy prior to HSCT [6], which was consistent with our findings. Likewise, malignancy relapse post-HSCT showed an expected numeric association with excess mortality, although significance was not reached. Finally, receipt of cord blood transplant appeared to be a detrimental factor, consistent with the reports of poor immune reconstitution post cord HSCT found in the literature [31,32,33].

Notably, we found that pre-HSCT surgical management of MCM showed a modest association with improved survival post-HSCT (although not statistically significant), which had also been suggested as a peri-transplantation strategy to prevent relapse of invasive fungal disease (not limited to MCM) [34]. This association had also been uncovered previously in several retrospective reviews of post-HSCT MCM and in patients with hematological malignancies and MCM without HSCT context [35,36]. This observation corroborates the significance of source control in MCM patients, including those with extrapulmonary lesions. Growing data in patients with invasive fungal sinusitis support that surgery is associated with improved survival if there is no intracranial involvement [37] (similar outcomes for *Aspergillus* and *Mucorales*) [38]. Moreover, complete surgical resection versus partial resection was reported to yield more favorable outcomes [39]. For pulmonary MCM, the literature indicates improved mortality with surgical management over medical management alone [40]. However, while prior retrospective studies confirmed surgical management as an independent predictor of favorable outcomes, our present analysis was insufficiently powered for multi-variable modeling. Therefore, this association is probably confounded by patient selection for surgery (e.g., patients with best performance status, no comorbidities and favorable prognosis of hematologic cancer).

All patients with data available had continued secondary post-HSCT prophylaxis (mainly with MCM-active triazole monotherapy) for widely variable time periods after HSCT, with decisions being fully individualized, although not always clearly outlined. In some cases, antifungals were continued only until engraftment [24,26], and in other cases, they were continued for 12 months post-HSCT or beyond that time guided by CD4 count thresholds [21]. This remains an area of debate as there is no clear guidance to determine duration of secondary prophylaxis, and patients on continued azole prophylaxis may experience antifungal-related adverse events that must be balanced with the risk of MCM recurrence. The low number of patients with MCM recurrence and inherent statistical biases precluded us from establishing associations between shorter duration of prophylaxis and increased risk of MCM recurrence or inferior survival outcomes. Our median of 3 months of secondary prophylaxis post-HSCT may be an appropriate starting point with prolongation of prophylaxis depending on the specifics of each case.

The optimal timing of HSCT relative to the initial diagnosis of MCM is usually informed by the severity and extent of infection, the degree of source control, and the urgency of transplantation for the underlying hematologic disease. The literature recommends treating patients for at least 4 weeks with documentation of some clinical, radiological, or microbiological response [33]. Other tests such as Mucorales-specific T-cell [41] or even NK responses [42] and positron emission tomography–computerized tomography might inform fungal control [43]. Our data set did not show any significant association between time from infection to HSCT and mortality but the analysis did not take into account the infection severity or the attributed cause of mortality.

None of the patients in our review had progression of MCM at the time of HSCT as they all achieved at least stable response to antifungal treatment. While we did not find significant differences in mortality between patients with stable response and those with partial or complete response, it is notable that none of the patients with complete response died. Interestingly, two published cases of patients with stable response reported the use of HSCT as salvage treatment not only for the hematological disease but also for MCM [7,13]. Both patients had favorable outcomes. However, despite these anecdotal cases, this approach does not seem feasible on a widespread basis and favorable reports might be subject to significant publication bias.

There is no conclusive difference in the published literature between mortality of human MCM after treatment with LAmB monotherapy versus combination therapy with MCM-active triazoles and a study with human subjects showed no significant difference with either posaconazole or isavuconazole used in combination with LAmB [44]. We did not find any significant impact on mortality in our study either, and disseminated MCM, although widely reported to have increased mortality compared to localized MCM [5], showed only a numerical but not statistically significant impact on mortality in our data set.

As a strength of our review, our analyses predominantly included probable/proven MCM diagnoses, avoiding challenges with the interpretation of possible MCM cases as often encountered in daily clinical practice. Only two patients had diagnoses only based on PCR without histopathological correlation. Although the dates of the included cases ranged from 1996 to 2025, 41% are from the past decade and 38% from the current decade, suggesting that they mostly reflect current practices.

The main limitation of our study is the undeniable publication bias in the literature reviewed. In particular, the concern of publication bias toward those with more favorable outcomes is underscored by the fact that the five cases from our institution had significantly worse outcomes than the published cases, with only two out of five patients surviving beyond 14 months post-HSCT. It is also important to consider that patients with severe MCM would be naturally excluded from evaluation for this review since they would likely not survive long enough to complete evaluation for HSCT in the first place. Additionally, the total number of cases available for review was relatively small. Combined with a lack of consistent reporting of key variables (e.g., GVHD management) and gaps in the reported data, this precluded the performance of multi-variable models to confirm independent predictors of survival outcomes post-HSCT or the assessment of associations specifically between MCM recurrence after HSCT and pre-HSCT variables. Thus, multicenter registries would be desirable to improve both reporting consistency and statistical power, minimize the publication bias, and provide more real-world evidence.

## 5. Conclusions

Combining a comprehensive review of the literature and five cases from our institution, we conclude that prior MCM should not be an absolute contraindication for HSCT, after careful consideration of both oncologic variables and MCM treatment response. Specifically, our data indicate that a suitable candidate for HSCT with a history of MCM is a patient with their primary malignancy in remission, continued on secondary antifungal prophylaxis for at least 3 months post-HSCT, and ideally with surgical intervention for source control pre-HSCT. However, the decision to move forward with HSCT needs to be individualized and consider other medical aspects beyond MCM.

## Figures and Tables

**Table 1:** Patient characteristics and outcomes.

Variable ^S1^ Unless Specified Otherwise, Numbers or Patients and Percentages (in Parentheses) Are Provided	All Patients	Death Within 12 Months Post-HSCT ^S1^		*p*-Value ^S1^
	(n = 29)	Yes (n = 5)	No (n = 20)	
Age (years), median (range)	44 (5–70)	42 (8–55)	43.5 (5–70)	0.472
Sex (male)	14 (48%)	2 (40%)	8 (40%)	0.776
Hematologic condition				
AML	20 (69%)	4 (80%)	13 (65%)	0.721
ALL	7 (24%)	1 (20%)	6 (30%)	0.974
Aplastic anemia	2 (7%)	0 (0%)	1 (5%)	0.902
Malignancy status pre-HSCT ^C1^				
Remission	10/27 (37%)	1 (20%)	6/19 (32%)	0.694
Induction/active	10/27 (37%)	0 (0%)	10/19 (53%)	0.199
Relapsed/refractory	7/27 (26%)	4 (80%)	3/19 (16%)	**0.031**
Antifungal prophylaxis before MCM diagnosis ^C2^				0.915
None	11/28 (39%)	2 (40%)	8/19 (42%)	
Non-MCM active	16/28 (57%)	3 (60%)	10/19 (53%)	
MCM-active	1/28 (4%)	0 (0%)	1/19 (5%)	
Infection site at MCM diagnosis				
Pneumonia (including sinopulmonary)	17 (59%)	1 (20%)	14 (70%)	0.146
Disseminated	9 (31%)	3 (60%)	5 (25%)	0.194
Other localized	3 (10%)	1 (20%)	1 (5%)	0.288
Modalities used to confirm MCM diagnosis ^C3^				
Biopsy/histopathology/cytology	21 (72%)	2 (40%)	15 (75%)	0.168
Culture	9 (31%)	3 (60%)	5 (25%)	0.240
PCR	7 (24%)	2 (40%)	4 (20%)	0.391
Mucorales Genus identified				
*Rhizopus* spp.	4/12 (33%)	1/3 (33%)	3/7 (43%)	0.818
*Lichtheimia* spp.	4/12 (33%)	1/3 (33%)	2/7 (29%)	0.909
*Rhizomucor* spp.	3/12 (25%)	1/3 (33%)	1/7 (14%)	0.617
*Mucor* spp.	1/12 (8%)	0/3 (0%)	1/7 (14%)	0.862
Initial antifungal therapy				0.673
Lipid AMB + MCM-active triazole	15 (52%)	3 (60%)	10 (50%)	
Lipid AMB monotherapy	14 (48%)	2 (40%)	10 (50%)	
Surgical therapy of MCM pre-HSCT ^C4^	22 (76%)	2 (40%)	17 (85%)	0.062
Any immunotherapy for MCM ^C5^	7 (24%)	1 (20%)	4 (20%)	1.000
Response to initial antifungal treatment ^C6^				0.799
Complete response	4/27 (15%)	0/4 (0%)	4/19 (21%)	
Partial response	14/27 (52%)	2/4 (50%)	10/19 (53%)	
Stable disease	9/27 (33%)	2/4 (50%)	5/19 (26%)	
Time (months) from infection to HSCT, median (range) ^C7^	3 (0–24)	5 (0–8)	3 (0–24)	0.607
Conditioning regimen ^C8^				0.518
Myeloablative	18/26 (69%)	3 (60%)	13/18 (72%)	
Reduced intensity conditioning	8/26 (31%)	2 (40%)	5/18 (28%)	
HSCT type				0.815
Allogeneic	26 (90%)	5 (100%)	18 (90%)	
Autologous	3 (10%)	0 (0%)	2 (10%)	
HSCT source ^C9^				
Peripheral	23/26 (88%)	3 (60%)	17/18 (94%)	0.072
Cord	2/26 (8%)	2 (40%)	0/18 (0%)	**0.019**
Bone marrow	1/26 (4%)	0 (0%)	1/18 (6%)	0.678
Haploidentical HSCT ^C9^	5 (17%)	1 (20%)	4/18 (22%)	0.711
Malignancy status post-HSCT ^C10^				0.165
Remission	19/24 (79%)	2/4 (50%)	16/19 (84%)	
Relapsed/refractory	5/24 (21%)	2/4 (50%)	3/19 (16%)	
Secondary antifungal prophylaxis post-HSCT				0.773
LAmB	14 (48%)	2 (40%)	10 (50%)	
MCM-active triazole	12 (41%)	3 (60%)	8 (40%)	
LAmB + MCM-active triazole	3 (10%)	0 (0%)	2 (10%)	
Duration (months) of secondary antifungal prophylaxis post-HSCT, median (range)	6.5 (1–26)	4 (1–9)	7 (1–26)	N/A ^S2^
GVHD ^C9, C11^	10/26 (38%)	2 (40%)	8/18 (44%)	0.986
High-dose GCS exposure	3 (10%)	0 (0%)	3 (15%)	0.218
MCM recurrence	5 (17%)	3 (60%)	2 (10%)	**0.016**
Possible	3 (10%)	3 (60%)	0 (0%)	
Probable/proven	2 (7%)	0 (0%)	2 (10%)	
Other clinically significant infection post-HSCT ^C12^	5 (17%)	1 (20%)	3 (15%)	0.633

**Statistical notes (S):** ^S1^ *p*-values were derived using Cox proportional hazards regression with Firth’s penalized partial likelihood (n = 28; 5 deaths, 20 survivors, and 3 patients censored due to loss of follow-up). One patient without follow-up duration was excluded. ^S2^ Not applicable. Bias due to shorter follow-up periods in deceased patients (i.e., time to death). **Clinical explanatory notes (C):** ^C1^ Patients with aplastic anemia excluded (not qualifying as a malignancy). ^C2^ One case report did not include information on primary prophylaxis. ^C3^ Many patients had more than one (positive) diagnostic method to confirm MCM (detailed in Table S1). ^C4^ A total of 8 patients had single lobectomy, 2 had more than one lobectomy, 1 had wedge lung resection that included resection of adjacent rib and affected pericardium, 1 underwent intrapericardial total extrapleural pneumonectomy, 5 had sinus debridement, 1 had surgical resection of a thigh lesion, 2 had radical surgical debridement of intrathoracic lesions, 1 had surgical drainage of cerebellar collection, and 1 had myringotomy and radical mastoidectomy. ^C5^ A total of 2 patients received granulocyte transfusions, 3 had G-CSF infusions, and 2 received both. ^C6^ Data not available for 2 patients. ^C7^ Data not available for 2 patients. ^C8^ Data not available for 3 patients. ^C9^ Only applicable to allogeneic HSCT recipients. ^C10^ Data not available for 3 patients. Patients with aplastic anemia also excluded. ^C11^ Considering overlapping sites, 9 patients had skin, 4 gastrointestinal, 1 liver, and 1 oral mucosal involvement. ^C12^ One patient with *Pseudomonas aeruginosa* bacteremia; one with *Rothia mucilaginosa* bacteremia, disseminated candidiasis, and BK virus cystitis; one with *Stenotrophomonas maltophilia* bacteremia and BK virus cystitis; one with *Enterococcus faecalis* muscle abscess and *Candida tropicalis* skin infection; and one with Epstein–Barr Virus viremia (causing post-transplant lymphoproliferative disorder).

## Data Availability

All relevant data are contained in the manuscript and Supplementary Materials.

## Supplementary Materials

The following supporting information can be downloaded at: https://www.mdpi.com/article/10.3390/jof12060423/s1, Table S1: Summary of clinical characteristics and outcomes of the 29 mucormycosis cases.

## Abbreviations

The following abbreviations are used in this manuscript:

MCM	Mucormycosis
HSCT	Hematopoietic stem cell transplant
NAIMI	Non-*Aspergillus* mold infections
GVHD	Graft-versus-host-disease
MDACC	MD Anderson Cancer Center
GCS	Glucocorticosteroid
AML	Acute myeloid leukemia
ALL	Acute lymphoblastic leukemia
PCR	Polymerase chain reaction
AMB	Amphotericin B
LAmB	Liposomal amphotericin B
CT	Computed tomography
BAL	Bronchoalveolar lavage
i.v.	Intravenous
MRI	Magnetic resonance imaging
rh-GM-CSF	Recombinant human granulocyte-macrophage colony-stimulating factor
G-CSF	Granulocyte colony-stimulating factor

## Appendix A. Four Additional Cases from MD Anderson Cancer Center

### Appendix A.1. First Patient (#1 in Supplementary Table S1)—2010

A 51-year-old Asian female with relapsed/refractory AML who had undergone prior treatment with PR-104 (experimental hypoxia-activated prodrug) and quizartinib was admitted with neutropenic fever and abdominal pain. Her initial CT abdomen revealed typhlitis and she was treated empirically with piperacillin-tazobactam with clinical improvement. Four days after admission, she again started experiencing high-grade fever and chills with new right upper quadrant pain and right shoulder pain. Repeat CT abdomen revealed improved typhlitis but a new 2.5 cm developing lesion concerning for liver abscess. She underwent aspiration of fluid, and the culture result was reported as zygomycetes. She had been on voriconazole prophylaxis previously and was switched to i.v. LAmB 5 m/kg/day, i.v. caspofungin 100 mg/day, and oral posaconazole 200 mg every 6 h. She continued LAmB for 18 weeks and caspofungin for 15 weeks; posaconazole was continued as secondary prophylaxis indefinitely.

Once her blastemia had decreased, the decision was made to proceed with matched sibling donor peripheral HSCT after myeloablative conditioning with busulfan, fludarabine, and clofarabine four months after her MCM diagnosis, and after radiological and clinical response to antifungal treatment. Her post-transplant course was complicated by MDR *Pseudomonas aeruginosa* bacteremia two months after transplant. She also had skin GVHD treated with topical corticosteroids. However, the patient had a relapse of her leukemia three months post-transplant.

She was hospitalized three months after her transplant with GI bleeding and bilateral pneumonia requiring intubation. Serum *Aspergillus* galactomannan was 1.11, and she was severely neutropenic with absolute neutrophil count (ANC) of 0. She received i.v. methylprednisolone for suspected GI GVHD and antimicrobial therapy with ceftazidime, trimethoprim-sulfamethoxazole, caspofungin, and LAmB. Days later, she experienced multiorgan failure (hepatic, renal, and pulmonary dysfunction) with extremely poor prognosis. Thus, the family decided to transition her to comfort care, and she passed away during that hospitalization. No autopsy was performed.

### Appendix A.2. Second Patient (#2 in Supplementary Table S1)—2019

A 31-year-old Hispanic female with relapsed B-ALL on cytarabine presented with pleuritic chest pain. CT chest revealed a left upper lobe cavitary nodule. BAL was non-diagnostic, but biopsy of the lung nodule revealed necrosis, acute inflammation, and fungal organisms consistent with Mucorales, so she was diagnosed with pulmonary MCM. Fungal culture had no growth.

She had been on isavuconazole prophylaxis but, due to concern for breakthrough infection, was switched to oral posaconazole 300 mg/day and i.v. AMB phospholipid complex 5 mg/kg/day. At the same time, she also underwent robotic thoracic resection of left upper lobe cavity. She completed 6 weeks of AMB and 7 weeks of posaconazole before the latter was switched back to isavuconazole due to liver function test abnormalities.

The patient had multiple reported CNS leukemia relapses but was in clinical remission at the time decision was made to proceed with HSCT three months after initial MCM diagnosis. She received myeloablative conditioning with busulfan, thiotepa, and fludarabine followed by matched unrelated donor peripheral HSCT with post-transplant cyclophosphamide.

Her post-transplant course was complicated by *Rothia mucilaginosa* bacteremia and BK virus hemorrhagic cystitis. Seven months after transplant, she was diagnosed with disseminated *Candida tropicalis* infection with CNS and cutaneous involvement, treated with AMB phospholipid complex and voriconazole. Thirteen months after transplant, she experienced a relapse of fungal pneumonia. Right lung biopsy revealed necrotic lung parenchyma and abundant fungal organisms morphologically favoring Mucorales. She was treated with caspofungin, AMB phospholipid complex, and posaconazole with improvement in oxygen requirement and fever but without radiologic and microbiological re-evaluation at the time.

One month later, she was re-admitted with small bowel obstruction thought to be secondary to leukemic infiltration as she was known to have extramedullary disease. At the time of her final hospitalization, she was not neutropenic but PET CT still showed extramedullary disease. She then decided to be transferred to home hospice and passed away at home 2 weeks later. No autopsy was performed.

### Appendix A.3. Third Patient (#3 in Supplementary Table S1)—2021

A 55-year-old Asian female with AML in remission on gilteritinib was transferred to our hospital after being admitted at an outside hospital with neutropenic fever and multifocal pneumonia. CT chest revealed reverse halo sign on the right lung, and CT abdomen revealed three suspicious liver lesions. She underwent liver biopsy showing fungal organisms with focal septation and angioinvasion suggestive of MCM but with negative immunohistochemistry for *Rhizopus* and *Aspergillus*.

She was on primary prophylaxis with voriconazole and was switched to i.v. LAmB 5 mg/kg/day for 1 month (interrupted for 2 weeks due to acute kidney injury), then switched to three times weekly. She also received isavuconazole 372 mg/day for 5 months and was then switched to oral posaconazole 300 mg/day for secondary prophylaxis. She had progression of solid nodules on CT chest 7 months after initial diagnosis of MCM, and BAL cytology was consistent with Mucorales. She was treated with posaconazole, LAmB, and caspofungin.

She was deemed appropriate for stem cell transplantation 8 months after initial diagnosis of MCM and underwent reduced intensity conditioning (RIC) with fludarabine, melphalan, total body irradiation, followed by haploidentical peripheral HSCT with post-transplant cyclophosphamide.

A month after transplant, during the same hospitalization, she had suspected engraftment syndrome briefly treated with i.v. methylprednisolone for two days. At the same time, she developed progressively worsening hypoxic respiratory failure. CT chest revealed left lower endobronchial lesion macroscopically concerning for MCM. BAL culture and cytology were negative for fungi. Secondary posaconazole prophylaxis was switched back to isavuconazole.

Six weeks after HSCT, she had experienced multiorgan failure attributed to disseminated MCM. The family’s decision was to transition her to comfort care and she passed away soon after. No autopsy was performed. During her final hospitalization, her leukemia seemed to have responded to the HSCT, and she was non-neutropenic.

### Appendix A.4. Fourth Patient (#5 in Supplementary Table S1)—2008

A 52-year-old white male with newly diagnosed chronic myelomonocytic leukemia/AML was hospitalized with neutropenic fever, cough, and diarrhea soon after initiation of induction chemotherapy with idarubicin and cytarabine. He was on primary antifungal prophylaxis with itraconazole and was initially treated empirically with meropenem and vancomycin, while itraconazole was switched to anidulafungin due to hyperbilirubinemia.

Ten days into his hospitalization, he developed left eye pain and swelling and CT sinus showed mucosal thickening. A few days later, he reported new diplopia, and repeat CT head showed orbital cellulitis. Ophthalmology reported concern for fungal invasion and CT chest revealed new nodules in the right lower lobe, and a solid 2.5 cm nodule in the left lower lobe. He was started on AMB lipid complex and posaconazole. He had neutrophil count recovery days after initiating active antifungal treatment and he was discharged home on i.v. AMB lipid complex 5 mg/kg/day, oral posaconazole 200 mg every 6 h, and i.v. anidulafungin 100 mg/day.

The patient had initially declined surgical intervention, but nearly two weeks after initiation of antifungal therapy, he underwent endoscopic sinus surgery with left maxillary antrostomy, left anterior and posterior ethmoidectomy, left sphenoidotomy, endoscopic removal of left inferior turbinate, and endoscopic removal of posterior septum. Surgical biopsy revealed numerous fungal organisms reported as zygomycetes. He then had surgical revision a week later with pathology revealing invasive fungal sinusitis. Anidulafungin was discontinued and he continued AMB lipid complex (intermittently held due to creatinine elevation), and posaconazole. He underwent another sinus debridement a month later and received a total of 3 months of AMB and continued posaconazole for secondary prophylaxis.

After two months of antifungal treatment, CT chest showed resolution of nodules but nearly 4 months after diagnosis, CT sinus revealed persistent sinus disease although the patient was asymptomatic and nasal endoscopy was not concerning for active sinusitis.

His leukemia was in remission, and 4 months after diagnosis of MCM, he underwent myeloablative conditioning with fludarabine and busulfan followed by matched-related donor peripheral HSCT.

His post-transplant course was complicated by skin GVHD a month after transplant, and then liver GVHD 7 months after transplant treated with photopheresis and methylprednisolone taper (continued for 5 months). He continued secondary antifungal prophylaxis for >12 months after transplant, albeit posaconazole was switched to caspofungin due to liver function abnormalities 9 months post-HSCT.

The patient was followed for 18 months post-transplant and showed no clinical evidence of recurrence of MCM. He remained in remission from his AML and with normal neutrophil count.
